# The minimal important difference in obsessive-compulsive disorder: An analysis of double-blind SSRI trials in adults

**DOI:** 10.1192/j.eurpsy.2024.1768

**Published:** 2024-09-20

**Authors:** Sem E. Cohen, Jasper B. Zantvoord, Taina K. Mattila, Bram W.C. Storosum, Anthonius de Boer, Damiaan Denys

**Affiliations:** 1 Department of Psychiatry, Amsterdam UMC, Amsterdam, The Netherlands; 2 Amsterdam Neuroscience Research Institute, Amsterdam, The Netherlands; 3 Medicines Evaluation Board, Utrecht, The Netherlands; 4 Arkin Institute for Mental Health, Amsterdam, The Netherlands; 5 Utrecht University, Utrecht, The Netherlands

**Keywords:** Obsessive-compulsive disorder, Double-blind randomized controlled trials, Pharmacotherapy

## Abstract

**Background:**

The change in symptoms necessary to be clinically relevant in obsessive-compulsive disorder (OCD) is currently unknown. In this study, we aimed to create an empirically validated threshold for clinical significance or minimal important difference (MID).

**Methods:**

We analyzed individual participant data from short-term, double-blind, placebo-controlled registration trials of selective serotonin reuptake inhibitors in adult OCD patients. Data were collected from baseline to week 12. We used equipercentile linking to equate changes in the Clinical Global Impression (CGI) scale to changes in the Yale-Brown Obsessive-Compulsive Scale (YBOCS). We defined the MID as the YBOCS change linked to a CGI improvement of 3 (defined as “minimal improvement”).

**Results:**

We included 7 trials with a total of 1216 patients. The CGI-scores and YBOCS were moderately to highly correlated. The MID corresponded to 4.9 YBOCS points (95% CI 4.4–5.4) for the full sample, or a 24% YBOCS-decrease compared to baseline. The MID varied with baseline severity, being lower in the group with mild symptoms and higher in the group with severe symptoms.

**Conclusions:**

By linking the YBOCS to the CGI-I, this is the first study to propose an MID in OCD trials. Having a clearly defined MID can guide future clinical research and help interpretation of efficacy of existing interventions. Our results are clinician-based; however, there is further need for patient-reported outcomes as anchor to the YBOCS.

## Introduction

Obsessive-compulsive disorder (OCD) is characterized by persistent thoughts (obsessions) and repetitive behaviors (compulsions). The global lifetime prevalence rate is 2% [1]. OCD is associated with an increased mortality risk, and without treatment, it may profoundly impair quality of life and social functioning [2]. Selective serotonin reuptake inhibitors (SSRIs) are currently the recommended pharmacological treatment for management of OCD [3, 4]. The gold standard for measuring OCD symptom severity is the Yale-Brown Obsessive-Compulsive Scale (YBOCS) [5]. This is a 10-item scale (symptom score range 0–40) with good validity and inter-rater reliability, which has assured its widespread utilization, in both clinical trials and clinical practice [6]. Currently, the magnitude of changes necessary to be relevant to patients or clinicians is unclear.

In addition to disease-specific scales, an overall impression of the patient’s well-being, as assessed by the clinician, is used in order to evaluate illness. The scales often used in clinical trials are the Clinical Global Impression Severity and Improvement scales (respectively, CGI-S and CGI-I) [7]. The CGI-S is a 7-point scale that ranks the assessor’s impression of illness severity, and the CGI-I is a 7-point scale that captures global improvement during treatment. CGI-scales rely on the subjective impression of the assessor-clinician and take into account all available, illness-specific information. Both CGI-S and CGI-I are valid, intuitive, and simple to use [8, 9].

In psychiatric research, the concept minimal important difference (MID) can be employed to evaluate the clinical effects of treatment. The MID refers to the change in symptoms necessary to bring about a relevant improvement for patients and/or clinicians, and was originally defined as the smallest difference in measured health status that signifies an important, rather than a trivial difference in patient symptoms [10]. There are multiple approaches to calculating the MID, one of which is anchoring a symptom-specific scale to the CGI by using equipercentile linking, facilitating deduction of the necessary change on a symptom scale in order to bring about one point improvement on the CGI [11, 12]. Equipercentile linking has been employed in multiple psychiatric illnesses, such as schizophrenia, bipolar disorder, and major depressive disorder [11, 13–15]. While definitions of “response” in OCD research (i.e., a YBOCS reduction of at least 35%), and a CGI-I score of 1 (“very much improved”) or 2 (“much improved”) are mainly consensus-based, equipercentile linking can be employed to add to an empirical foundation for these response definitions [16]. To the best of our knowledge, no study has yet linked the YBOCS to the CGI-S and CGI-I, which might give new perspectives on the clinical efficacy of OCD treatments.

In this study, we set out to link the CGI to the YBOCS using short-term double-blind, placebo controlled trials of SSRIs in patients with OCD. SSRIs are commonly used for OCD, with a small (to medium) effect size when tested in double-blind randomized controlled trials [17, 18]. Our primary goal was to find the MID, which we defined as three points on the CGI-I (“minimally improved”). As a secondary outcome, we report the YBOCS change linked to a CGI-I score of 2 (“much improved”). Furthermore, we linked the YBOCS scores to the CGI-S at baseline, as well as to the change in CGI-S during treatment.

## Methods

For this study, we used individual participant data obtained from short-term, randomized, placebo-controlled efficacy trials with SSRI’s. These studies were submitted to the Dutch Regulatory Authority (the Medicines Evaluation Board, MEB) in order to obtain marketing authorization. Trial data were shared with the MEB under the condition that names of the compounds would not be released and original publication would not be cited. We only included studies in adult patients (18 years and older) using both YBOCS and CGI measures. We used outcomes from the active and placebo treatment arms.

The YBOCS is a 10-item psychometric test, with five items specifically on obsessions and five items specifically on compulsions. Each item can be rated from 0 to 4 points (4 being worst), which means that assigned scores range from 0 to 40. The YBOCS is used to score baseline severity and improvement (or deterioration) during treatment. The CGI-S captures the assessor’s interpretation of the patient’s current illness with the following scores: 1 = normal (not at all ill), 2 = borderline mentally ill, 3 = mildly ill, 4 = moderately ill, 5 = markedly ill, 6 = severely ill, and 7 = extremely ill. The CGI-I captures the assessor’s impression of change (positive or negative), with the following scores: 1 = very much improved, 2 = much improved, 3 = minimally improved, 4 = no change, 5 = minimally worse, 6 = much worse, and 7 = very much worse. We used biweekly measurements of the CGI-I from weeks 2 to 12, and we predefined a CGI-I score of 3 as a measure for MID, as this equals “minimally improved” [19–21]. In addition to the CGI-I, we used biweekly measurements of the CGI-S and YBOCS from weeks 0 to 12.

Our primary outcome was defined as the YBOCS change score (YBOCS score at follow-up minus YBOCS at baseline) that was linked to a CGI-I score of 3. Our secondary outcomes were the CGI-I 2 (“much improved”) and CGI-S scores linked to the YBOCS at baseline, as well as the CGI-S change score (CGI-S at follow-up minus CGI-S at baseline) linked to the YBOCS change score.

We first tested our hypothesis that the CGI and YBOCS were correlated by calculating the Spearman correlation coefficients for each time point, and we predefined a correlation of 0.5 or higher as sufficient to perform equipercentile linking.

In order to find the MID, we searched for the corresponding psychometric points on the YBOCS and CGI-I/-S by equipercentile linking [12]. We calculated percentile rank functions for the CGI and YBOCS variables and performed equipercentile linking with pre-smoothing using the log-linear smoothing method to address the potential issues of sparse data in extreme score ranges. To evaluate the stability and variability of our linking results, we applied a bootstrapping approach with 500 resamples [22, 23]. This process allowed us to generate confidence intervals. We then visualized our results for our primary and secondary analyses using a plot depicting the relationship between the linked scores.

Because OCD symptom change depends on baseline severity in SSRI RCTs, it is likely that the clinical relevance of symptom reduction does, as well [24]. Therefore, we included percentage YBOCS reduction in our analysis. Furthermore, to account for this dependence, we employed a post hoc analysis linking the CGI-I to patients from different severity groups using established severity benchmarks (YBOCS 14–21 for mild symptoms, 22–29 for moderate symptoms, and 30–40 for severe symptoms) [25]. We did so as absolute point reduction is more easily clinically interpretable compared to percentage change.

The analysis was conducted using the R programming language, specifically employing the equate package for equipercentile linking [26, 27]. We preregistered our analysis plan at the Open Science Forum (https://doi.org/10.17605/OSF.IO/JEM7V).

## Results

We included 7 trials with a total of 1216 patients with CGI-S and YBOCS scores at baseline. Then, 635 patients were prescribed an SSRI and 510 a placebo, and 122 patients were included for randomization but did not receive medication due to early attrition. They were included in our baseline measures and excluded from the follow-up measures. Mean YBOCS severity at baseline was 24.5 (±4.9 SD), which is indicative of severe OCD; mean age was 37.9 (±12 SD) [28]. A total of 46% of the included patients were female. We found no baseline differences between the treatment and placebo groups. For an account of baseline variables, see Table 1. During follow-up, a decline in the number of patients is visible, with the most sudden drop between weeks 10 and 12, as not all studies had a 12-week follow-up (week 2 N = 1224, week 4 N = 1160, week 6 N = 1110, week 8 N = 1054, week 10 N = 984, and week 12 N = 650; see Supplementary Table S1).

**Table 1.** Patient characteristics at baseline

	Placebo	Active treatment	Test for between-group differences
*N*	510	706	
*N* Gender *F (%)*	223 (44)	335 (48)	χ^2^ = 1.5 (p 0.22)
*N* Gender *M (%)*	287 (56)	371 (52)	
Mean age (SD)	37.7 (12)	38.0 (12)	t = −0.38 (p 0.70)
Mean YBOCS (SD)	24.6 (5.0)	24.4 (4.8)	t = −0.57 (p 0.57)
Mild *N*	141	182	
Moderate *N*	294	429	
Severe *N*	74	95	
Median CGI–S (IQR)	5 (1)	5 (1)	W = 150908 (p 0.51)

*Note*: *p* = probability value after t-test for numerical values (age, YBOCS), χ^2^ for dichotomous outcomes (gender) and Mann–Whitney U test (W-statistic) for categorical variables (CGI-S). N = number, SD = standard deviation, YBOCS = Yale-Brown Obsessive-Compulsive Scale, CGI-S = Clinical Global Impression – Severity. “Mild” = YBOCS 14–21, “Moderate” = YBOCS 22–29, “Severe” = YBOCS 30–40, according to empirical benchmarks by Cervin et al. [25].

CGI-I for each week was correlated with YBOCS change scores (Spearman’s correlation ranging between 0.64 and 0.82 with an increase every week, for a full table of correlation scores; see Supplementary Table S2). Equipercentile linking showed that a YBOCS reduction of 4.9 points (95% CI −5.5 to −4.4, corresponding to a 24% YBOCS reduction) was linked to a CGI improvement score of 3 (“minimally improved”). After splitting the sample according to severity, the “mild” group had an MID of – 3.1 (95% CI −4.2 to −2.1), the “moderate” group had an MID of 5.1 (95% CI −5.8 to −4.4) and the “severe” group had an MID of 7.2 (−9.1 to −5.3) (see Supplementary Table S5).

**Figure 1.**
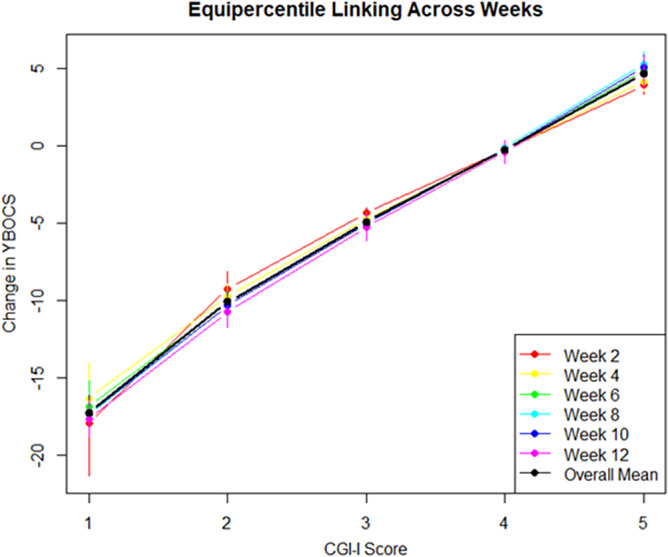
Equipercentile linking of CGI-I and change in YBOCS score, per week. Black line = mean linking for each week. CGI-I = Clinical global impression scale - improvement, YBOCS = Yale-Brown Obsessive Compulsive Scale

Furthermore, a CGI-I score of 2 (“much improved”) corresponded to a 46% YBOCS reduction compared to baseline (95% CI −56 to −36), or an average 10 point reduction (95% CI (−11 to –9.4). Table 2 and Figure 1 show the linked results of the remaining CGI-I and YBOCS scores. Since the weekly number of patients with a CGI-I score of 6 or 7 was low, we did not calculate linking scores for these values (see Supplementary Table S1 for number of patient per CGI per week).

**Table 2.** Change in YBOCS linked to change in CGI-I

CGI-I	YBOCS change (95% CI)	YBOCS % change (95% CI)
1	−17 (−19 to −16)	−83 (−97 to −70)
**2**	−10 (−11 to −9.4)	−**46 (−56** to **−36)**
**3**	**−4.9 (−5.4** to **−4.4)**	−24 (−42 to 6.5)
4	−0.26 (−0.66 to 0.13)	−1.1 (−9.3 to 7.0)
5	4.5 (3.8–5.3)	21 (12–29)

*Note*: Mean scores for weeks 2, 4, 6, 8, 10, and 12. YBOCS change in absolute point difference and in percentage change. 95% CI = 95% confidence interval (upper – lower), CGI-I = Clinical Global Impression Scale – Improvement.

**Figure 2.**
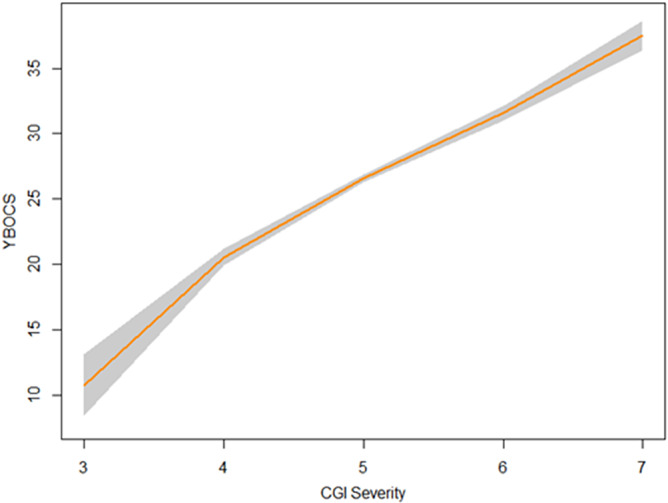
Equipercentile linking of CGI-S and YBOCS at baseline. CGI - S = Clinical global impression scale - severity, YBOCS = Yale-Brown Obessive Compulsive Scale.

At baseline, YBOCS and CGI-S were moderately correlated with a Spearman correlation coefficient of 0.62 (see Supplementary Table S2). After equipercentile linking, a CGI-S score of 4 (moderately ill) corresponded with a YBOCS of 21 (95% CI 20–22) (Table 3, Figure 2). Patients who were scored markedly ill at baseline (CGI-S of 5) had an equated baseline YBOCS of 26 (95% CI 26–27). CGI-S 6 and CGI-S 7 were equated with, respectively, a YBOCS of 31 and 38 (95% CI 31–32, 95% CI 36–39). A baseline of CGI-S 3 was linked to 10 points on the YBOCS (95% CI 7.8–13).

**Table 3.** Change in YBOCS linked to one point CGI-S change at baseline

CGI severity	YBOCS score	95% CI lower	95% CI upper
3	10	7.8	13
4	21	21	22
5	26	26	27
6	31	31	32
7	38	36	39

**Figure 3.**
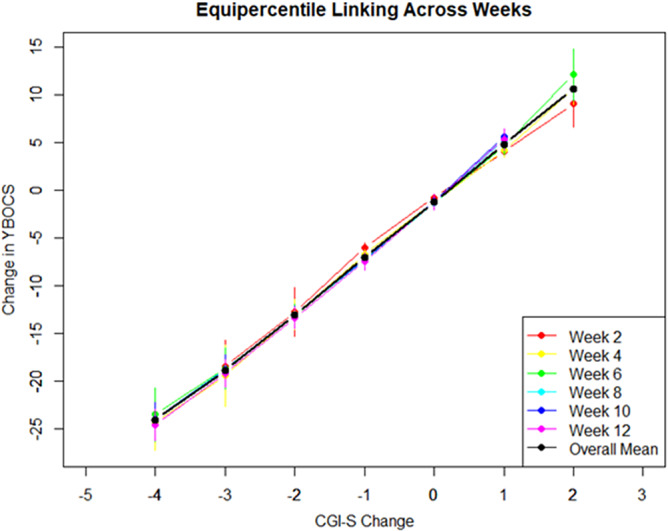
Equipercentile linking of CGI-S change and YBOCS change, per week. Black line = mean linking for each week. CGI - S = Clinical global impression scale - severity, YBOCS = Yale-Brown Obessive Compulsive Scale.

Equipercentile linking of the change in CGI-S scores compared to baseline showed a difference of 5.3–6.4 points on the YBOCS per CGI-S point (see Table 4 and Figure 3). Correlation coefficients between CGI-S change scores and YBOCS were moderate to strong from week 4, increasing from 0.58 to 0.78 (see Supplementary Table S1).

**Table 4.** Change in YBOCS linked to one point difference in CGI-S Change

CGI-S change	YBOCS change	95% CI upper	95% CI lower
−4 to −3	5.3	2.4	8.0
−3 to −2	5.8	3.3	8.4
−2 to −1	6.0	4.5	7.5
−1 to 0	5.8	5.0	6.6
0–1	6.1	5.0	7.0
1–2	6.4	4.1	8.7

*Note*: Mean scores for week 2, 4, 6, 8, 10, and 12. A negative CGI-S change score means an apparent clinical improvement compared to baseline.

## Discussion

In this study, we for the first time developed an MID for adults with OCD in placebo-controlled RCT’s, in order to create an empirically validated threshold for clinical significance. By linking YBOCS change to minimal improvement according to the CGI-I, we found a general MID of 4.9 points on the YBOCS.

We further showed that the MID is dependent on baseline symptom severity. In patients with higher baseline symptom scores, a more substantial decrease in symptoms is necessary to bring about a noticeable change, relative to less severely ill patients. Using percentage symptom reduction, which incorporates differences in baseline severity, we found that a YBOCS decrease of about 25% corresponded with minimal improvement, which corresponds to what has been defined as a threshold for partial response for OCD [29].

Using data registries from the Medicines Evaluation Board, we were able to use a large sample of high-quality registration studies to develop an MID for obsessive-compulsive disorder. As most clinical OCD studies, SSRI trials use symptom reduction measured with the YBOCS as outcome, which is the primary efficacy measure recommended by regulatory agencies [30]. A statistically significant difference on a continuous outcome scale between active treatment and placebo is used to test clinical efficacy. Our study shows the smallest observable difference, or MID, for clinicians (4.9 YBOCS points) to be larger than the 3.5-point treatment effect found in short-term efficacy trials on SSRIs in OCD [31]. This discrepancy between the MID and the average improvement in OCD mirrors the literature on SSRIs for major depressive disorder, where multiple studies on a variety of scales found an MID that exceeds results from placebo-controlled trials [32].

Both at baseline and during follow-up, we also found that one point change in the CGI-S is consistent with five to six point changes in the YBOCS. Even though the CGI-S and CGI-S-change scores were not predefined as signifying MID, these results further illustrate that a minimum improvement of five YBOCS points is necessary in order to bring about a change in symptoms that is noticeable in the clinical setting.

Aside from an MID, we found that a CGI-I score of 2 points (“much improved”) was linked to a YBOCS reduction compared to baseline of 46%. This is higher than the commonly used definition of clinical response as 35% YBOCS reduction [33]. Consistent with our results, a study using a different methodological approach and different patient populations found that solely using the 35% YBOCS reduction criterion would lead to a overestimation of response by about 2%, compared to combining the YBOCS and CGI-I [29]. This is further supported by findings from a retrospective analysis showing that a YBOCS reduction between 40 and 50% optimally predicts CGI-I 1 or 2 after OCD treatment [34]. As recently stated in a consensus paper, an expert-based definition of response in OCD is operationalized as 35% clinical reduction *plus* a CGI-I score of 1 or 2 [16]. Our findings support this operationalization, indicating that in clinical practice, relying solely on a 35% cut-off score on the YBOCS for defining response may be too lenient, potentially resulting in the undertreatment of patients.

An MID might act as an empirically informed measure for the interpretation of judging overall effects that are found in a trials (placebo-controlled, head-to-head) or cohorts. However, as this pertains group-level outcomes, individual clinical response within trials varies widely and a group effect smaller than that of the MID does not exclude the possibility of patients having an adequate treatment effect. Regarding clinical research, the MID might be applied as a dichotomous outcome, similar to *response* or *remission.* This would identify the chance of minimal improvement after intervention compared to placebo and could be used in addition to the chances of response/remission. As such, it could also be employed for clinical decision-making, by informing patients regarding the effects of the intervention.

However, there are some caveats regarding interpretation of the MID. For instance, we have shown that the MID is dependent on baseline severity, and thus, the overall outcome cannot be assumed to be relevant for every patient. Other baseline criteria, such as gender or age, might also influence the size of the MID [35]. When linking the CGI to the YBOCS, we still rely only on symptom-related outcome measures. A recent transdiagnostic survey showed that when estimating the clinical status of the patients, CGI-S assessors rely more on symptom scales and clinical interviews, and less on staff observation or non-symptom-specific patient-perspective health outcomes [36]. None of the trials available used standardized questionnaires focusing on patient recovery and/or quality of life. To our knowledge, only one short-term double-blind SSRI trial has used such a scale as an outcome measure [37]. Non-illness-specific scales of disability of quality of life would be an important addition to future clinical trials on OCD pharmacotherapy. In fact, novel clinical trials focusing on interventions such as Deep Brain Stimulation have included these questionnaires [38]. Furthermore, the CGI was designed as a clinician-rated instrument; thus, linking the CGI with another clinician-rated instrument makes for an assessor-based MID. A patient-centered MID would require linking symptom scores to patient-reported outcome measures (PROMs) [39]. The individual participant data that were made available to us did not include these outcomes. Reevaluation of all double-blind RCT’s on SSRI’s included in a recent systematic review from our research group shows that only four studies used some form of patient-reported severity scale, namely the patient-rated global impression scale [31, 40–43]. Together, these findings emphasize the paucity of PROMs in double-blind SSRI trials in OCD.

Another important element of developing a patient-centered MID would be to directly involve mental health service users and/or lived experience groups [44]. For instance, a recent OCD psychotherapy trial has predefined their MID as five YBOCS points [45]. They did so after consulting a Lived Experience Advisory Panel, who determined that a mean 0.5 point reduction on all 10 YBOCS items might be considered clinically meaningful. Even though the authors did not use direct empirical analysis and the interventions differed, their proposed minimum for clinically significant change resembles our findings.

Our study has some shortcomings. We only included double-blind randomized controlled trials in which the included sample and clinical setting might have biased our results. For example, in the real-world clinical setting (as opposed to the RCT-setting), an assessor might have known the patient for a longer time or might have spoken with the patient’s family or spouse, which could influence how the CGI-I or CGI-S are scored. Since all included studies were SSRI trials, generalizability to other interventions, such as psychotherapy or neuromodulation might be limited. Furthermore, CGI-I and CGI-S measurements do not take into account side effects when determining improvement in health status, as they instruct assessors not to incorporate side effects [46]. Considering side effects with SSRI treatment for OCD are common, it is to be expected that MID’s would increase when also taking into account adverse drug reactions [47].

Notwithstanding the limitations of our study, using equipercentile linking of the CGI-I and YBOCS, we were able to propose an MID of 4.9 YBOCS points for the pharmacological treatment of OCD. This finding sheds new light on the efficacy of existing pharmacological interventions and can guide future clinical research. To better inform shared decision-making between patients and clinicians when managing OCD, future pharmacotherapy trials must prioritize the use of patient-reported outcomes and quality-of-life measures.

## Supporting information

Cohen et al. supplementary material Cohen et al. supplementary material

## Supplementary material

The supplementary material for this article can be found at http://doi.org/10.1192/j.eurpsy.2024.1768.
