# Supplementary material for: The minimal important difference in obsessive-compulsive disorder: An analysis of double-blind SSRI trials in adults
Source: Eur Psychiatry. 2024 Sep 20;67(1):e53. doi: 10.1192/j.eurpsy.2024.1768 (PMC11457115; doi:10.1192/j.eurpsy.2024.1768)
Supplement: Cohen et al. supplementary material [file S0924933824017681sup001.docx]

**Minimal important difference supplementary material**

| **Week** | **CGI-I 1** | **CGI-I 2** | **CGI-I 3** | **CGI-I 4** | **CGI-I 5** | **CGI-I 6** | **CGI-I 7** | **Total n** |
| --- | --- | --- | --- | --- | --- | --- | --- | --- |
| **2** | 11 | 64 | 379 | 656 | 102 | 11 | 1 | 1224 |
| **4** | 27 | 123 | 426 | 472 | 96 | 16 | 0 | 1160 |
| **6** | 40 | 191 | 398 | 390 | 80 | 11 | 0 | 1054 |
| **8** | 62 | 230 | 350 | 341 | 65 | 5 | 1 | 1110 |
| **10** | 77 | 253 | 292 | 300 | 56 | 6 | 0 | 984 |
| **12** | 91 | 184 | 181 | 163 | 30 | 5 | 0 | 650 |
| **Week** | **CGI-S 1** | **CGI-S 2** | **CGI-S 3** | **CGI-S 4** | **CGI-S 5** | **CGI-S 6** | **CGI-S 7** | **Total n** |
| **2** | 2 | 8 | 88 | 541 | 434 | 141 | 10 | 1224 |
| **4** | 5 | 25 | 114 | 521 | 358 | 127 | 10 | 1160 |
| **6** | 10 | 33 | 179 | 457 | 322 | 105 | 4 | 1054 |
| **8** | 11 | 58 | 201 | 437 | 260 | 84 | 3 | 1110 |
| **10** | 10 | 75 | 210 | 412 | 207 | 69 | 1 | 984 |
| **12** | 9 | 80 | 168 | 221 | 127 | 45 | 4 | 650 |

**Supplementary table S1:** Amount of patients for each CGI-I and CGI-S score, per week

| **Week** | **CGI-I – YBOCS change** | **CGI-S change – YBOCS change** | **CGI-S – YBOCS** |
| --- | --- | --- | --- |
| **Baseline** | *NA* | *NA* | 0.62 |
| **2** | 0.64 | 0.40 | 0.67 |
| **4** | 0.70 | 0.58 | 0.70 |
| **6** | 0.74 | 0.68 | 0.76 |
| **8** | 0.79 | 0.71 | 0.80 |
| **10** | 0.77 | 0.73 | 0.81 |
| **12** | 0.82 | 0.78 | 0.86 |

**Supplementary table S2:** Spearman’s rank correlation coefficient per week **.**

| **Week** | **CGI-S-change -4** | **CGI-S-change -3** | **CGI-S-change -2** | **CGI-S-change -1** | **CGI-S-change 0** | **CGI-S-change 1** | **CGI-S change 2** |
| --- | --- | --- | --- | --- | --- | --- | --- |
| **2** | 0 | 6 | 79 | 285 | 1011 | 82 | 4 |
| **4** | 1 | 18 | 79 | 348 | 830 | 74 | 2 |
| **6** | 3 | 37 | 113 | 387 | 664 | 73 | 5 |
| **8** | 9 | 53 | 146 | 393 | 539 | 71 | 0 |
| **10** | 12 | 58 | 177 | 373 | 496 | 69 | 1 |
| **12** | 14 | 57 | 161 | 251 | 289 | 34 | 1 |

**Supplementary table S3:** Amount of patients for each CGI-S-change score, meaning CGI-S score minus CGI-S at baseline. Theoretically the scores range from -6 to +6, but -5,-6 and 3,4,5,6 are not present in the dataset.

| **CGI improvement** | **YBOCS change** | **95% CI lower** | **95% CI upper** |
| --- | --- | --- | --- |
| 1 | 6.8 | 4.8 | 8.9 |
| 2 | 5.3 | 4.3 | 6.2 |
| **3** | **4.7** | 4.0 | 5.4 |
| 4 | 4.6 | 3.7 | 5.5 |

**Supplementary table S4 :** Change in YBOCS linked to one point difference in CGI-I, mean scores for week 2, 4, 6, 8, 10 and 12. CGI-I of 3 is our primary outcome and corresponds to a YBOCS change of 4.7

| **CGI improvement** | **YBOCS change** | **95% CI lower** | **95% CI upper** |
| --- | --- | --- | --- |
| 1 | - 14.1 | -17 | - 12 |
| 2 | - 8.4 | - 9.4 | - 7.3 |
| **3** | - 3.7 | - 4.5 | - 2.9 |
| 4 | 0.79 | - 0.031 | 1.6 |
| 5 | 6.0 | 4.6 | 7.3 |
| 6 | 11.8 | 9.0 | 14.6 |

**Supplementary table S5** CGI-I for the group of patients with a mild to moderate baseline severity, equaling a Yale-Brown Obsessive-Compulsive Symptom score of less than 24.

| **CGI improvement** | **YBOCS change** | **95% CI lower** | **95% CI upper** |
| --- | --- | --- | --- |
| 1 | - 19.3 | - 21.5 | - 17 |
| 2 | - 12.5 | - 14 | - 11 |
| **3** | - 5.8 | - 6.6 | - 5.0 |
| 4 | - 0.9 | - 1.5 | - 0.4 |
| 5 | 3.1 | 2.3 | 3.9 |
| 6 | 7.5 | 5.8 | 9.1 |

**Supplementary table S6** CGI-I for the group of patients with a severe to extreme baseline severity, equaling a Yale-Brown Obsessive-Compulsive Symptom score of equal or more than 24.
